# Barriers and attitudes influencing non-engagement in a peer feedback model to inform evidence for GP appraisal

**DOI:** 10.1186/1472-6920-12-15

**Published:** 2012-03-23

**Authors:** Esther Curnock, Paul Bowie, Lindsey Pope, John McKay

**Affiliations:** 1 Department of Postgraduate General Practice Education, NHS Education for Scotland, 2 Central Quay, Glasgow, Scotland, G3 8BW, UK

## Abstract

**Background:**

The UK general practitioner (GP) appraisal system is deemed to be an inadequate source of performance evidence to inform a future medical revalidation process. A long-running voluntary model of external peer review in the west of Scotland provides feedback by trained peers on the standard of GP colleagues' core appraisal activities and may 'add value' in strengthening the robustness of the current system in support of revalidation. A significant minority of GPs has participated in the peer feedback model, but a clear majority has yet to engage with it. We aimed to explore the views of non-participants to identify barriers to engagement and attitudes to external peer review as a means to inform the current appraisal system.

**Methods:**

We conducted semi-structured interviews with a sample of west of Scotland GPs who had yet to participate in the peer review model. A thematic analysis of the interview transcriptions was conducted using a constant comparative approach.

**Results:**

13 GPs were interviewed of whom nine were males. Four core themes were identified in relation to the perceived and experienced 'value' placed on the topics discussed and their relevance to routine clinical practice and professional appraisal: 1. Value of the appraisal improvement activity. 2. Value of external peer review. 3. Value of the external peer review model and host organisation and 4. Attitudes to external peer review.

**Conclusions:**

GPs in this study questioned the 'value' of participation in the external peer review model and the national appraisal system over the standard of internal feedback received from immediate work colleagues. There was a limited understanding of the concept, context and purpose of external peer review and some distrust of the host educational provider. Future engagement with the model by these GPs is likely to be influenced by policy to improve the standard of appraisal and contractual related activities, rather than a self-directed recognition of learning needs.

## Background

Since 2003, general practitioners (GPs) in the United Kingdom (UK) have been contractually obliged to engage in a peer appraisal of their professional practice [1]. This involves an externally nominated peer colleague (appraiser) reviewing specific aspects of an individual GP's (appraisee) professional practice on an annual basis. Feedback to aid professional development is provided in a 'non-judgemental' and formative manner. The appraisal interview remains a confidential interaction between the appraiser and appraisee, unless issues arise during discussions that give cause for serious concerns about the well-being of the doctor or patients.

There is an expectation that the combined evidence from five consecutive annual appraisals would substantially inform a proposed UK system of revalidation for all medical practitioners [2]. However, a recent White Paper [3] has declared that this is no longer a viable option because the existing appraisal process is thought to lack the robustness, objectivity and clarity which are necessary to support a high stakes revalidation system. Additionally, the evidence for the effectiveness of this form of peer appraisal in improving individual learning and the quality of patient care remains inconclusive [4].

It is now clear that appraisal will have to move from mere engagement to a demonstration of both learning for the doctor and impact on patient care. By necessity, therefore, the process must become more *transparent and objective *[5]. Peer review and feedback either by the appraiser or through other external 'quality control' mechanisms is likely to become more prominent in deciding on whether the evidence supplied for appraisal is of a sufficient standard to inform revalidation.

One suggested method of addressing these criticisms is through the external verification of specific aspects of a GP's work. In the west of Scotland medical deanery, a peer feedback model [6,7] has been established for over a decade and has been proposed as one method to 'add value' to the current Scottish GP appraisal system. The underlying principles of the model are based on an adaptation of cognitive continuum theory [8], which has been described in related published work 6. GPs in the deanery are currently able to voluntarily submit evidence of three core appraisal activities (clinical audit, significant event analysis (SEA) and consultation technique via videotaped consultations) for external review by trained peer colleagues. A small financial cost is levied for this service. This type of approach may be of a much wider interest and potential value beyond the regional deanery. Details of the educational purpose, method and theoretical origins underpinning this specific peer feedback model have been published previously [6,7,9].

It is proposed that this peer feedback model may help to resolve the 'appraisal dilemma' by providing some *objective *evidence of professional development and performance activities which are necessary to strengthen the existing model in support of revalidation [6,7]. Furthermore, it would present an opportunity to review whether the activities undertaken by the GP are of a desired educational standard and, where necessary, allow for informed feedback to improve the quality of the submitted work.

The empirical evidence underpinning the acceptability, feasibility and educational impact of the peer feedback model is moderate and growing steadily [9-17]. However, although a significant proportion of GPs has voluntarily participated in some aspect of the model, a clear majority in the deanery has yet to do so. It is unclear why many choose to forgo this potentially valuable learning opportunity, while others are prepared to voluntarily engage. In order to further strengthen engagement with the peer feedback model, we aimed to explore the views of GPs who had not yet participated. We were specifically interested to identify potential barriers to engagement in the per feedback model (beyond the financial cost) and ascertain attitudes to the concept of external peer review in the current context of appraisal and planned revalidation.

## Methods

The research was qualitative in design. Semi-structured, in-depth interviews with individuals were used to explore understanding, knowledge, attitudes and practicalities of engaging in and receiving external peer feedback. Initial exploratory interviews with six GPs were conducted by LP in 2009. The themes emerging from these interviews formed the basis of the topic guide for the interviews held in 2010. These interview data were not included in this study as too much time had elapsed and the interviewer had taken up another research position. An iterative study design [18] was employed in order to allow emerging themes to be explored with greater depth in subsequent interviews. Adopting this reflexive approach reduces the risk of making unwarranted assumptions about perspectives on peer feedback in the sample.

### Setting, sample and sampling procedure

The research was conducted in the West of Scotland general practice setting, across five geographical health boards. In the UK GPs have completed vocational training and are licensed to provide a range of primary health care to individuals and families. A database review of 3059 GPs working in the region found 1923 (63%) who had not engaged with the model. The 'non-engagers' group was stratified by three inclusion criteria (Table 1) to ensure a range of perspectives were captured - gender, type of GP position held, and postgraduate training practice status. This stratified the population into twelve groups for sampling. Sample sizes relative to the total group were calculated to result in a sample of 100 potential study participants. Random selection methods were used to select individuals from each group. A letter was sent to the sample of 100 explaining the study purpose, with an invitation to participate in a confidential semi-structured interview lasting approximately 60 minutes. Of the 29 who replied seven expressed an interest in participation. A second round of invitations was sent to a further sample of 100 GPs using the same process, which elicited 24 positive responses with a further six GPs able to attend interviews. A total of thirteen GPs were interviewed.

**Table 1:** Study inclusion criteria, variables and rationale

Inclusion criterion	Variable	Rationale for inclusion
**Gender**	Female or male	The GP workplace is often and conveniently sub-divided into common groupings because these may contain individuals with differing perspectives. The study attempted to reflect this in part by ensuring that the viewpoints of individual GPs from each of the 3 different groups were adequately represented.
	
**Postgraduate training practice status**	Training or non-training status	
	
**Type of GP position held**	GP principal*	
		
	Sessional doctor+	
		
	Other (e.g. Salaried GP)$	

* A registered, vocationally-trained medical practitioner who is contracted by a local health authority to take unsupervised responsibility for patient care (personal and general medical services)

^+^A fully qualified general practitioner who works sessions (half or full day) rather than having a contract with a local health authority

^$ ^A fully qualified general practitioner who works on an employed basis with a practice that is contracted with a local health authority or out-of-hours service

### Data collection

Interviews were conducted by EC at a venue chosen by each participant. A semi-structured prompt guide was used to steer discussion on the following areas: understanding of peer review and feedback; exposure to peer feedback activities; factors affecting participation in peer feedback (perceptions of barriers and facilitators). Interviews lasted 45 minutes on average, ranging from 30 to 60 minutes. The interviews were audio-recorded with permission, transcribed and anonymised. Formation of theory based on the emerging themes was explored in more depth with subsequent participants until saturation was achieved. The interviewer reflected on her perspectives of each interview and made contemporaneous notes in a comments sheet. This was shared and discussed with research colleagues in order to deepen the understanding of the topic and stimulate further thinking.

### Data analysis

Each interview recording was listened to thoroughly to check for inaccuracies in transcription. Thematic analysis of the interview transcriptions was conducted by a constant comparative approach [19]. Sections of meaningful text were identified within each interview independently by two researchers. The sections of text were coded; codes were then compared with each other looking for similarities, differences and general patterns to form categories, which were then compared across interview transcripts to identify themes. The categorisation changed several times during this process. Particular attention was paid to negative cases (cases inconsistent with the emerging analysis), which were used to clarify themes during analysis discussion sessions.

### Ethical approval

The study proposal was pre-screened by NHS Lothian MREC 'A' committee for research ethical approval but was judged to be service evaluation.

## Results

Of the thirteen GPs interviewed (nine male, four female), ten were GP principals, two were sessional doctors and one was a salaried GP. Study participants worked in a mixture of urban and rural practices across the west of Scotland with a spectrum of patient list-sizes. Six participants were involved in delivering either under-graduate or post-graduate training, and five were members of the regional educational programme which permitted free access to the peer feedback model (Table 2).

**Table 2:** Characteristics of Study Participants: Personal, Professional & Educational (n = 13)

1. Personal characteristics	
1.1 Gender	
Female	4
Male	9

**2. Professional characteristics**	

2.1 GP position	
GP principal	10
Sessional doctor	2
Salaried GP	1

2.2 Geographical location	
Rural	4
Urban	7
Mixed	2

2.3 Years qualified as doctor	Range = 5-35 years
	Mean = 21 years

2.4 Membership of 'The Partnership' (regional continuing professional development scheme)	
Yes	5
No	8

2.5 Practice list size (number of patients)	
2000 to 5999	6
6000 to 10000	2
> 10000	2
N/A	3

**3. Educational characteristics**	

3.1 Involvement in Training	
None	7
Medical students	2
Hospital doctors-in-training	2
Specialty Training	2

Four core themes were identified in relation to the barriers and attitudes influencing engagement in the peer feedback model in the current context of appraisal and planned revalidation:

1. Value of the improvement activity (i.e. audit, SEA, consultation technique)

2. Value of external peer review

3. Value of the peer feedback model

4. Attitudes to external peer review (including appraisal)

A summary of the key barriers and attitudes related to each theme is outlined in Table 3.

**Table 3:** Summary of key barriers and attitudes by theme

Theme	Barriers	Attitudes
• Value of the improvement activity (i.e. audit, SEA, consultation technique)	• Relevance of activity to professional practice and every day work	• Activities are generally helpful, but not to all participants specifically
		
		• Some believe their own improvement methods are sufficient

• Value of external peer review	• Variation in understanding and purpose of peer review	• More challenging than internal review by close colleagues
	
	• Viewed as more 'formal' and 'selection and control'	• Standard and validity of work enhanced
		
	• Informed internal colleagues' views just as valid	

• Value of the peer feedback model	• Constrained by defined, structured formats	• Perceived as overly formal and additional work
	
	• Other peer review options available	• Formalisation seen positively with regard to link with appraisal and regulation

• Attitudes to external peer review (including appraisal)	• Distrust and suspicion of host organisation	• Face-to-face peer feedback more valuable

	• Lack of awareness of model	
		
	• Practical barriers to engagement	

### Value of the improvement activity

There was disagreement amongst participants as to the inherent professional or clinical value of all three improvement activities offered for peer review. Although most GPs could understand why these specific activities could enhance professional practice, others viewed some or all of them as not having a direct relationship to improving their day-to-day clinical work and so questioned their importance to them.

"*Audit I am sure does improve systems, does raise the standards of personal practice but maybe not as, not as imminently as looking at a significant event or looking at yourself doing a consultation. Those are far more in your face, you know far more personal; they are far more likely to bring around a change in practice or a reflection about what you are doing*" (GP2)

"*They are some easily measurable things... they are measurable but I don't know that that necessarily reflects good pract*ice" (GP1)

For some participants even though they acknowledged that the improvement activities discussed could enhance aspects of professional practice generally they did not perceive that it had or would lead to direct changes in their own practices specifically. They were confident in their own assumptions about the sufficiency of their experiences and abilities as a means to self-improve and keep up-to-date and did not appear highly motivated to participate in these specific improvement activities.

"*It is highly unlikely at this stage that I am massively going to change my approach or change my style of operating which has clearly worked reasonably okay up to now*" (GP4)

### Value of external peer review

It was recognised that GPs can be exposed to many different models or styles of external peer review often without a full realisation that they are doing so. For some this was revealed in a limited and possibly conflicting understanding of the context, meaning and purpose underpinning peer review as an educational construct. For example, several GPs understood peer review to relate primarily to 'multi-source feedback' for which they displayed strongly negative feelings. Although this was not wholly accurate it nevertheless led to a biased perception of what 'peer review' entailed for them.

There was recognition amongst many participants that the possibility of external review of their professional activities by peers would be more 'challenging' than 'internal' review from GP partners or associates. For many this was thought to be clearly beneficial as it would enhance the validity of their work because it was perceived to be more objective and formalised - particularly if the peer reviewer was not known to the GP. In addition, it may offer a demonstration to any interested external authorities, or even their own patients, that their professional performance was of a sufficient standard.

Conversely, the concept of external review was thought by others to offer little additional benefit either as a quality assurance tool or as an educational activity. This was particularly so if study participants' immediate GP work colleagues had recognised educational expertise and knowledge that may be associated with being a professional appraiser, specialty training supervisor or holding a medical regulatory position with a relevant local or national organisation. Additionally, the notion of independent, external review was also regarded as "*more formal review*" which in turn was associated with "*selection and control*" aspects of regulatory medical assessment, rather than as a formative and developmental educational exercise.

"...*the person who would be more detached, could look at the situation more objectively that is probably a good point also, again they wouldn't be biased by knowing you, probably more objective and that would probably be the best advantage I think really" (GP7)*

"*One of our partners is a CHCP *[Community Health Care Partnership] *chairman so he is usually ahead of changes in the game, we have got two appraisers... our Practice Manager is a QOF *[Quality & Outcomes Framework] *visitor... so we have lots of people who are on the ball learning things you know from the point of view of our practice*" (GP4).

### Value of the peer feedback model

The external peer feedback model was thought by many to be constrained by the defined, structured formats to be adhered to when performing and submitting the improvement activities under discussion. The act of complying with these formats was thought to miss the important nuances of daily professional practice that influenced contextual and performance issues and could only be fully appreciated and understood by those who worked in and experienced that particular environment.

"...*the disadvantage of external feedback would have, it wouldn't it would only have what it saw on paper whereas internally the people can have a feel for what you are doing and how you are doing it, so its finer details but it is also sort of the 'wishy-washy' more difficult to define aspect of what you have done and how you have done it, only internal people would be able to pick up on*." (GP12)

For some GPs their internal working environment was thought to offer numerous opportunities for 'robust' peer review from a range of clinical and also managerial colleagues. They therefore perceived very little advantage in engaging with the external peer feedback model, the preparation for which they thought to be unnecessary, overly formal and added workload.

"A*t our meetings in the surgery alone there is probably about thirty or forty folk at it... so it's a pretty good appraisal in itself... within the practice I think there is enough people that we get pretty good feedback*" (GP10)

"*I cannot be bothered with all that extra bits because I don't need it... and well God ok, that I haven't looked at everything but I just haven't sat down and written it all bit by bit... but people will argue, 'well if you don't do it properly it is just sloppy, la la la la', fine, I don't need the formalization anymore thank you very much*" (GP9)

In contrast, those GPs who could foresee a benefit in the peer feedback model did so because of the perceived advantage that could be gained through the process of formalising the external review of aspects of their professional performance for regulatory reasons, rather than the potential educational and developmental impact which may be accrued.

"*I daresay one of the benefits value wise will be strengthening my appraisal and revalidation folder in terms of ticking all the right boxes there*" (GP6)

### Attitudes to external peer review (including appraisal)

A lack of professional trust in the true purpose and potential integrity of both the external peer feedback model and the national appraisal system was clearly evident for a significant minority of participants. Previous experience of high stakes assessment processes as part of specialty training led to some expressing negative attitudes to participation in a formative external feedback system. For others a willful non-participatory attitude was formed because of a perception that the system is 'disciplinary' in nature, or is potentially so, or is simply not to be trusted because of the healthcare role of the organisation which hosts the peer feedback model (the national body responsible for the education and training of the healthcare workforce in Scotland):

"*I ended up having to do a second audit [for summative assessment during GP training] having failed one on almost a technicality. I think that may have put me off the process...I didn't really go through a positive feedback process*" (GP4)

"*Big Brother... I don't see it as helpful at all, I don't see anything that suggests from anything that I have been involved in to suggest that they are actually trying to help*..." (GP6)

For others, external review was thought of more favourably if it was considered to be 'true peer review' which was associated with face-to-face feedback. This type of feedback was considered to be much more valuable than the existing system of providing a personalised written report summarising reviewers' developmental comments on the standard of the improvement activity undertaken.

"...*it certainly would be useful to know that that somebody was working in General Practice and not maybe somebody who was you know in a ivory tower as an academic GP you know, and wasn't you know and didn't have their feet on the ground sort of thing*" (GP10)

"...*but I think verbal feedback is useful, without verbal feedback (the written report) I think it would be worse because I think with verbal there is interaction, any misunderstandings could be rectified then*" (GP9)

A range of marketing and resource perceptions about barriers to non-engagement were also identified such as a lack of awareness of the peer review system; time to participate, financial cost and equipment constraints; and unfavourable geographical locations (where potential participants felt isolated from the central co-ordinating office for peer review). These perceived pragmatic difficulties were raised by, and on behalf of, sessional and out-of-hours doctors by participants. These particular peripatetic GP groups were thought to have greater challenges to overcome when arranging to video-tape consultations or undertake clinical audits before submitting this evidence for peer review.

"...*the difficulty with them [video-taped consultations] is I suppose the timing and the amount of work required..." *(GP3)

"*it is not my practice, I am there as an employee, hired locum for that session, and so obviously before I think before I would be sending it off I would need to clear that with the Partners and other people involved before I sent it out to an external source*" (GP1)

Current experiences of the national appraisal system also influenced attitudes to peer review and whether to proactively engage with the separate peer feedback model. Most participants recognised the existing appraisal system as a form of external peer review. However, for some, experiences of this system were associated with a perceived lack of learning and improvement opportunities in relation to the time and resource invested, while for others this form of external peer review was slightly more challenging but had a minimally positive impact overall.

"...*so far I have had three appraisers, and if that is what an external person offers thank you - good night...the last guy was slightly better, the first two were a waste of space... so that has not encouraged outside support*" (GP7)

## Discussion

This small study represents the first steps in trying to determine why GPs may forgo potentially important learning opportunities that could strengthen their portfolio of evidence for professional appraisal. Preparedness by GPs in this study to participate in the external peer feedback model to enhance appraisal activities involves three interlinked themes: the value to the practitioner of the improvement activity itself; the perceived benefits of external review and feedback; and the professional esteem in which the provider of the peer review is held. The themes represent a hierarchy whereby ultimate participation depends on acceptance of the value of the first or subsequent theme. These 'conditions of value' are modified by the participants' attitudes to the national appraisal system, the educational model of external peer review under study and the status of the host organisation.

### Findings in context

The finding that GPs must first of all value the improvement activity offered as part of the peer review process is consistent with aspects of adult educational theory which suggests that learners often undertake activities that they conceive as being of personal interest and highly relevant to their daily professional practice [20].

The evidence base for the perceived value of the three core appraisal improvement activities outlined in terms of impact on professional practice and patient care remains moderate [21-23]. However, two of three activities (SEA and clinical audit) are proposed as key elements of enhanced GP appraisal and revalidation which implies that regulatory authorities place a high value on their role as important indicators of professionalism and performance [24,25]. Some study participants are clearly sceptical about the effectiveness of such activities and the 'opportunity-cost' of participation, which is of potential concern. However research on the peer review system has highlighted variation in knowledge, attitudes and abilities, while also demonstrating the often critical importance of these activities to making healthcare safer, improving professional performance and enhancing the quality of services [10-14].

Some participants also appear confused about what constitutes 'peer review' which may be understandable given the variety of related initiatives described in the literature, many of which would struggle to truly reflect any mainstream definitions of 'peer review' [26]. For example, some GPs associated peer review only with 'multi-source feedback', which involves a range of colleagues not all of who are 'peers'. Paradoxically these GPs are highly likely to be exposed to a multitude of external peer review activities such as prescribing reviews, training practice accreditation visits, contractual verification visits and annual GP appraisal - but did not associate these professional obligations with the concept of 'peer review'. The importance of understanding the terminology goes beyond 'semantics' given the professional role of revalidation and appraisal, and also the advent of the peer review of prescribing patterns and specialist referrals recently introduced into general medical service contracts [27].

External peer review was seen by some as having the potential to enhance the validity of their professional practice, which is consistent with findings from those GPs who have actually taken part in the educational models offered to participants, including GP appraisers [6,9,10]. Conversely, others did not see the value or thought it limited, which reflects previous findings for external peer review through the Scottish GP appraisal system [4].

Some participants felt external peer review has little to offer over internal feedback from immediate work colleagues in strengthening appraisal evidence. This may represent a 'cognitive dissonance' [28], whereby conflicting ideas about the purpose and value of peer review are held simultaneously. Equally, for some, where internal review mechanisms are thought to be robust (either because of work colleagues' special interests, outside appointments or the variety of feedback options offered) external review may indeed be of limited benefit, although this will remain speculative without evidence to the contrary. We should acknowledge that these internal colleagues (as well as GP appraisers), although potentially informed, are not 'trained' to review and feedback on the standard of clinical audits, significant event analyses or consultation performance.

We know that many practitioners submit their audits and SEA reports for external peer review only for confirmation that this submission evidence for appraisal will be judged by trained colleagues to be of a satisfactory standard or otherwise [9]. It is possible that if one member of the GP team has demonstrated a perceived competence in a particular area then the value of external review for others may be diminished if both a 'benchmark' and local guidance are already available within the practice.

It is unsurprising that the host organisation which manages the peer feedback model is in itself a barrier to engagement. Research suggests that professionals, where possible, may seek to avoid submissions to, or contacts with, administrative bodies which have a governance or regulatory remit over them [9,10]. Previous experience of dealing with the organisation may have led to reluctance to engage voluntarily with it, particularly if that experience was negative (e.g. refusal of training practice accreditation or a poor GP appraisal encounter). This distrust by some about potentially exposing elements of their professionalism via the peer feedback model is interesting as previous research about hosting a hypothetical reporting system for patient safety incidents found that this organisation was the option overwhelmingly preferred by GPs compared with other national healthcare bodies listed [29].

For some participants, the role of GP appraisal is seen as enforcing unnecessary bureaucracy into an already busy job with little or no educational gain [4]. To try and enhance appraisal with more 'peer judgments' is, for these GPs, even less appealing. Additionally there may be an underlying resentment to participate in and provide evidence for appraisal, which may be seen by them as a slight on the professionalism of GPs individually and as a group [9,10]. This contrasts with previous research which suggests that GPs are acutely aware of the need for appraisal to evolve in support of revalidation in order to ensure and maintain the confidence of patients and doctors [30].

A recent literature review suggested that peer review [26] is a common element of performance assessment and is sometimes "...*the only mechanism to judge the professional practice of others working in the same field...because the peer regularly performs similar work and possesses the relevant expertise to evaluate it*". A fundamental irony is that the GP reviewers who are part of the peer feedback model described are the ones who have undergone the training to gain the '*expertise*' necessary to '*evaluate*' and '*judge*' the elements of '*professional practice*' that are under scrutiny from an appraisal (and regulatory) perspective - rather than the GP appraisers themselves. Study participants were largely aware of this but were still reluctant to engage with the model because of a combination of professional matters, pragmatic issues and attitudinal beliefs that all questioned the 'value' in exposing aspects of their professionalism on an independent, external and verifiable basis. The corollary is that it is unknowable if these aspects of professionalism are indeed exposed to some form of internal review by work colleagues or are subject to self-assessment, which recent evidence suggests is of limited value [31].

### Strengths and limitations of the study

Study strengths included interviews being undertaken with GPs at a range of career stages, working in different practice contexts (training & non-training), holding different status positions (principals, sessional and salaried doctors) with a mix of experience and roles outside of general practice, all of which is reflective of working arrangements in this setting. The interviewer (EC) was a doctor at an early stage in her career. This was perceived to have been an advantage particularly with the older GPs who may not view her to be part of the educational establishment. The interviewer strived to maintain an open attitude to the data; collaboration with a second researcher at the data analysis stage helped in this respect.

Limitations included the small number of study participants and their voluntary status which may have skewed the group towards those that already had a prior interest in the subject. Although we took great care to stratify potential GP recruits depending on the type of position they held, ultimately of those who were able to attend the interviews the great majority were GP principals. The interviews allowed in-depth discussion with individuals; although saturation was reached it is possible a bigger study would provide further insights than those gained here. The sample size also limited the ability to judge where most GPs sit along the process i.e. which of the judgements on value are most critical to most GPs. A reticence to share openly was perceived amongst younger GPs, who may have more closely identified the interviewer with the organisation which hosts the peer feedback model and is responsible for delivering most aspects of education and training in the region. The use of a semi-structured interview guide may have resulted in topics being missed, however an iterative approach to the content of the interview guide throughout the period of interviewing and analysis helped reduce this limitation.

## Conclusions

The study identified a range of important barriers and attitudes influencing non-engagement in the peer feedback model. How engagement with the model is increased from this 'non-engager' group is highly likely to be influenced by the decisions of local and national policy-makers, rather than a self-directed recognition of learning need by these GPs or a targeted 'promotional-push' by the host organisation. Future engagement levels will be dependent on how seriously decision-makers wish to 'add value' to the appraisal process or, for example, in quality assuring the standard of significant event analyses undertaken as part of the general medical services contract with health authorities (which contains a financial inducement for this element). There are, however, potential feasibility challenges (e.g. peer reviewer training and funding support) to be overcome if the peer feedback model is to be implemented on a wider scale. At present there appears to be a lack of potential alternatives other than this approach or the status quo.

## Competing interests

PB and JM are educational researchers who contribute to the design, management and evaluation of the peer review model under study and are also directly employed by the host organisation.

## Authors' contributions

EC and LP helped to design the study, carried out the interviews, and analysed the data. EC drafted the initial manuscript. JM assisted with data analysis and contributed to drafting the manuscript. PB conceived of the study, acquired funding, participated in its design and coordination and helped to draft the manuscript. All authors read and approved the final manuscript.

## Pre-publication history

The pre-publication history for this paper can be accessed here:

http://www.biomedcentral.com/1472-6920/12/15/prepub
